# Arthroscopic autologous chondrocyte implantation in the hip for the treatment of full-thickness cartilage defects: A case series of 29 patients and review of the literature

**DOI:** 10.1051/sicotj/2017037

**Published:** 2017-12-19

**Authors:** Steffen Thier, Christel Weiss, Stefan Fickert

**Affiliations:** 1 Sportchirurgie Heidelberg, ATOS Clinic Heidelberg 69115 Heidelberg Germany; 2 Institute of Biostatistics, University Medical Centre Mannheim, Medical Faculty Mannheim, University of Heidelberg 68167 Mannheim Germany; 3 Sporthopaedicum Straubing Berlin Regensburg 94315 Straubing Germany; 4 University Medical Centre Mannheim, Medical Faculty Mannheim, University of Heidelberg 68167 Mannheim Germany

**Keywords:** Hip arthroscopy, Autologous chondrocyte implantation, ACI, Cartilage defect, Matrix-associated autologous chondrocyte implantation, MACI

## Abstract

*Purpose*: Current literature indicates that the appropriate treatment of articular cartilage defects has significant influence on the postoperative outcome after hip arthroscopy. In the hip, arthroscopic treatment of cartilage defects is technically challenging, especially the autologous chondrocyte implantation/matrix-associated autologous chondrocyte implantation (ACI/MACI) procedures. The purpose of this prospective study was to introduce two injectable MACI products with self-adherent properties. Furthermore, we report short-term outcome and review the current literature.

*Methods*: Full-thickness cartilage defects of 29 patients caused by the femoroacetabular impingement (FAI) were treated arthroscopically with an injectable MACI product in a two-step surgical procedure. The patient-related outcome was assessed with International Hip Outcome Tool (iHOT33), Euro-Quol group score (EQ-5D) and Non-Arthritic-Hip-Score (NAHS) at baseline, six weeks, six, 12 and 24 months.

*Results*: Twenty-nine out of 46 patients (27 male/two female) with a mean age of 30.3 years (range 18–45 years) and an average defect size of 2.21 cm^2^ were available for follow-up after a mean of 19 months (range 6–24 months). All defects were located on the acetabulum International Cartilage Repair Society (ICRS) grade 3A–3D (nine 3A; eleven 3B; six 3C; three 3D). Twenty-six patients had associated labral pathology (23 repair 1–5 anchors; three partial trimming). Twenty-seven defects were caused by the FAI (20 CAM, six combined, one Pincer), two cases were of traumatic cause. An overall statistically significant improvement was observed for all assessment scores at an average follow-up of 19 months.

*Conclusion*: In this study, we present short-term data of new arthroscopic injectable matrix-associated, autologous chondrocyte implants as a treatment option for full-thickness cartilage defects of the hip. All patient-administered assessment scores demonstrated an increase in activity level, quality of life and reduction of pain after a 19-month follow-up. Further randomized controlled trails (RCTs) with comparison of natural history, bone marrow stimulation techniques and MACI of the hip have to approve the results in long-term follow-up.

## Introduction

The femoroacetabular impingement (FAI) is one of the main causes of hip cartilage defects that may subsequently lead to the development of hip osteoarthritis. The CAM-type FAI is particularly associated with large acetabular cartilage defects [1]. As in the current literature there seems to be a correlation between the stage of cartilage loss and postoperative outcome, the appropriate treatment of articular cartilage defects has significant influence on the postoperative outcome [2–5].

Articular cartilage is a tissue with unique capacity of load distribution and low-friction articulating surfaces [6]. Despite approximately four million load cycles a year with peak loads around 18 MPa, the articular cartilage is able to maintain its biomechanical function over decades [7]. Unfortunately in adults, the low metabolic and proliferative activity of articular cartilage results in a limited intrinsic capacity of self-repair [8].

Several surgical techniques, such as marrow stimulation techniques, osteochondral transplantation, autologous matrix-induced chondrogenesis (AMIC) and autologous chondrocyte implantation (ACI), have been made to restore articular surfaces. These techniques are routinely used in the knee. In the hip however, arthroscopic treatment of cartilage defects is technically challenging, mainly because of the restricted space in the central compartment. Consequently, most studies of the current literature report data on the microfracture procedure. These studies are mostly case series without control groups and rather small patient numbers classified as evidence-based medicine (EBM) levels III–IV [2, 3, 9–13].

In contrast to the knee joint, only limited data has been published on arthroscopic or open autologous chondrocyte transplantation in the hip [14–17]. The main reasons for the limited use of ACI procedures on the hip are the restricted space during arthroscopy due to osseous and labral containment and the technically demanding procedure [14]. Additionally, there are high demands on the ACI-System, which should be capable of a purely arthroscopic implantation without further fixation with sutures, adhesives or resolvable nails [14–16]. The development of third generation ACI, so-called matrix-associated chondrocyte implantation, combines self-adhesive properties with purely arthroscopic implantation [18–20].

The purpose of this case series is to report short-term data of two different injectable matrix-associated autologous chondrocyte implantation (MACI) products for the arthroscopic treatment of cartilage defects of the hip joint. The postoperative outcome is measured by using established scoring systems (International hip outcome tool (iHOT33), Non-arthritic-hip-score (NAHS), Euro-Quol group score (EQ-5D)). The results of these products are evaluated on the basis of the present reviewed literature.

## Materials and methods

This prospective case series was carried out according to the principles of the Declaration of Helsinki. We report early clinical data of 46 patients with isolated acetabular cartilage defects treated with an arthroscopic, matrix-associated autologous chondrocyte implantation (MACI) in a two-step procedure.

The preoperative patient assessment consisted of a clinical examination and standardized conventional X-rays. Anterior-posterior and cross-table radiographs [21] were performed and evaluated for signs of FAI and osteoarthritis.

Furthermore, all patients underwent magnetic resonance arthrography (MRA) with additional radial reconstruction for the detection of chondral and labral damage. Solely patients with suspicion of an acetabular cartilage defect in the MRA were eligible for inclusion [22].

After inclusion and exclusion criteria were verified and the patient provided informed consent, hip arthroscopy was performed. If a cartilage defect grade 3A–4 according to the International Cartilage Repair Society (ICRS) score were detected, the cartilage was harvested and blood samples were taken for the cultivation process.

Our hospital is authorized for the withdrawal, donation and procurement of human tissues and cells in accordance with the Directive 2004/23/EC. The study was approved by the Local Ethics Committee.

### Inclusion criteria

Twenty-nine patients (27 male/two female) fulfilled the inclusion criteria with an age between 18 and 50 years. Patients with an acetabular full-thickness cartilage defect scored 3A–4 according to ICRS and a minimum follow-up of six months were included.

### Exclusion criteria

Patients with non-contained acetabular cartilage defects, damage to the subchondral bone lamella in the defect, multiple defects or kissing lesions (opposing defects) were excluded. Furthermore, all patients with a Kellgren and Lawrence score for osteoarthritis > 2 and signs of dysplasia were excluded.

### Outcome evaluation

Baseline evaluation was done before index hip arthroscopy. Further outcome evaluations were performed at six weeks, three, six, 12 and 24 months after ACI utilizing three validated scores: EQ-5D, (general health/mental and physical); iHOT33 (pain, hip-specific function, daily living and sports); NAHS (sport activities, function) [23–25].

### Aim of study

The aim of the study was to compare the subjective patient outcome at 19 months after ACI of the hip with the baseline evaluation before arthroscopy by utilizing the NAHS, iHOT 33 and EQ-5D.

### Operation technique and treatment with NOVOCART^®^ Inject and Chondrosphere^®^

Hip arthroscopy was performed with the patient placed in the supine position. Approximately 10–15 mm joint distraction was used. To access the central compartment the anterolateral and anterior portal were established. Subsequently, the hip joint was investigated for labral and chondral damage. After appropriate classification of the cartilage defect according to ICRS, the indication for treatment with ACI was verified. Treatment of coexisting pathologies was performed during index arthroscopy (e.g., labral repair, offset reconstruction).

As many as 2–3 full-depth cartilage cylinders were harvested from the head-neck junction during first arthroscopy.

In cases of NOVOCART^®^ Inject, the cartilage specimens and 10 mL autologous patient serum were sent to the manufacturer. Patient’s chondrocytes were isolated from the cylinders and expanded as a primary culture in vitro in a Good Manufacturing Practice (GMP) approved facility (TETEC AG, Reutlingen, Germany) [18, 20].

In cases of Chondrosphere^®^, the cartilage specimens and 200 mL autologous patient serum were added to co.don (Fa. co.don AG, Teltow, Germany) for further isolation and culturing of three-dimensional spheroids as previously described [26].

**Figure 1.**
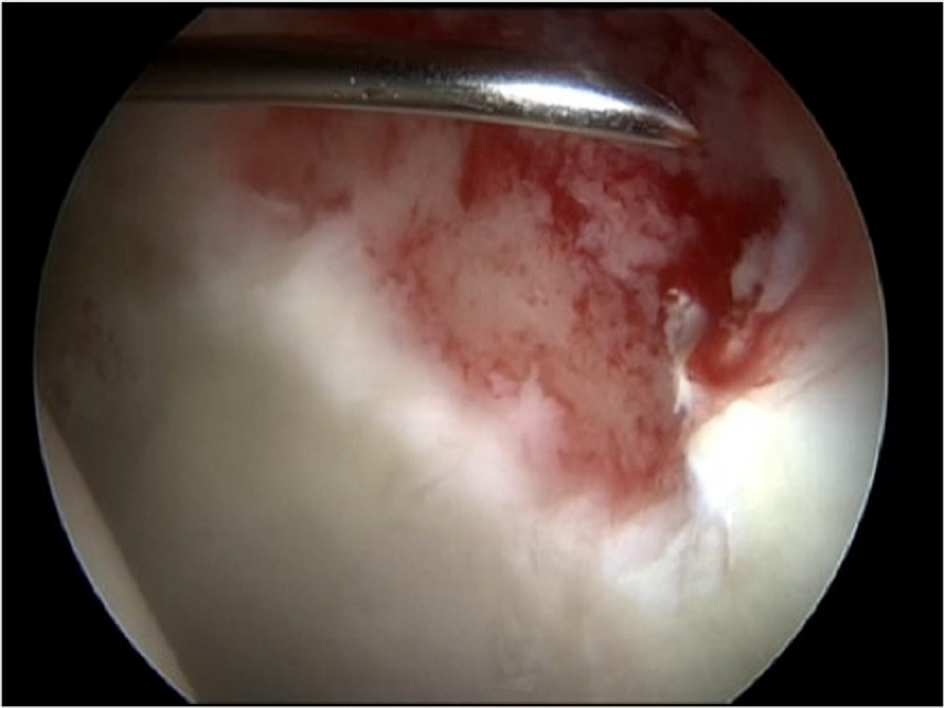
Adjustment of the application needle after debridement of a full-size cartilage defect at the anterolateral acetabulum.

In the second procedure, the defect was debrided arthroscopically to produce stable perpendicular margins (Figure 1). An additional posterolateral portal was established to drain intraarticular fluid. Fluid irrigation was then stopped to achieve dry conditions in the joint. Depending on the angulation, MACI was implanted through the anterior or anterolateral portal.

**Figure 2.**
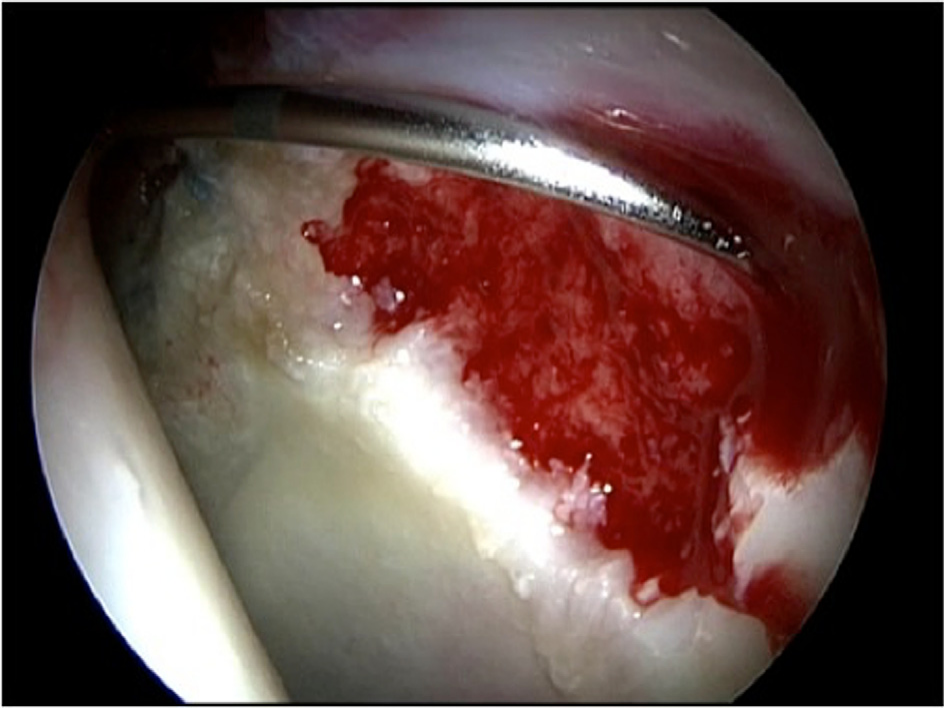
Application of NOVOCART® Inject.

**Figure 3.**
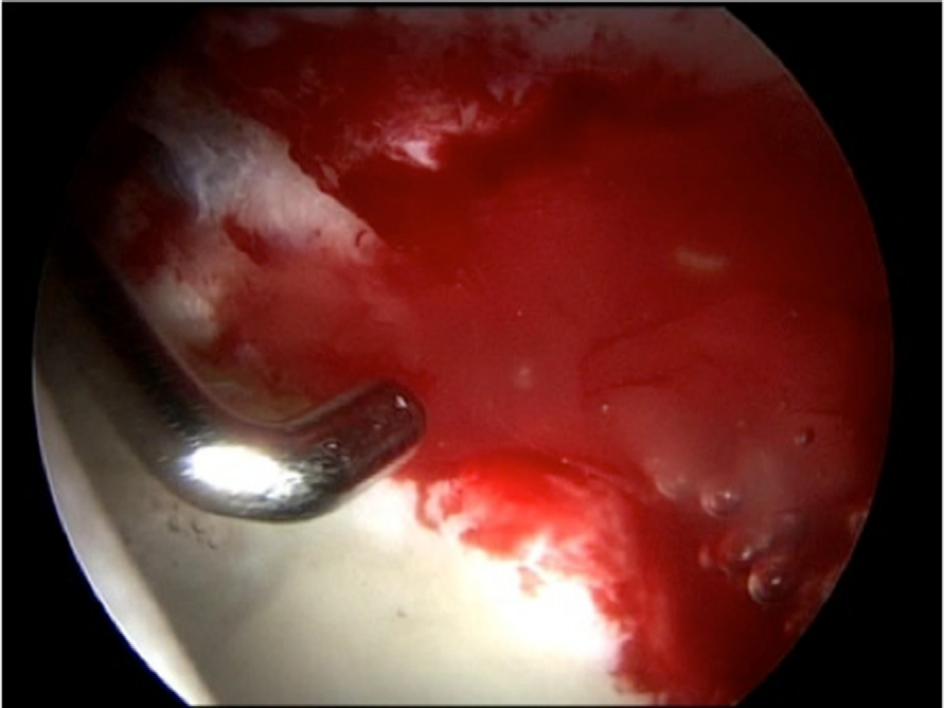
Testing the stability of matrix-associated autologous chondrocyte implant (MACI) with a hook after application and fluid irrigation.

The chondral defect was carefully covered with NOVOCART^®^ Inject, a combination of autologous cartilage cells and an in situ polymerizable hydrogel, through the deformable applicator (Figure 2). The injectable hydrogel, a combination of human albumin and hyaluronic acid, together with the autologous chondrocytes polymerizes in 30–60 s and bonds immediately to the bottom of the defect (Figure 3).

**Figure 4.**
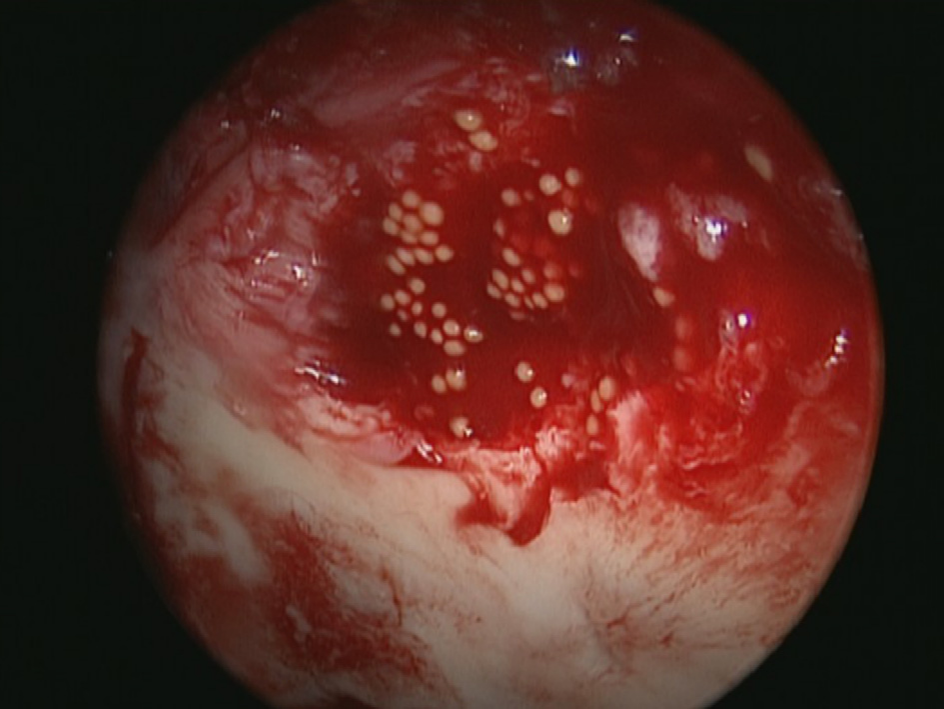
Application of Chondrosphere^®^.

The Chondrosphere^®^ implantation is performed in a similar fashion. A deformable applicator, which can be adjusted in length and angle, is used for the dropwise implantation of the spheroids in the defect (Figure 4). Immediately after the introduction of the spheroids their distribution can be adapted with a hook.

The spheroids bond after approximately 20 min is macroscopically visible when the spheroids change their form from round to flat.

Due to the adhesive properties of the MACI-Systems no further fixation is needed.

### Rehabilitation

After index surgery, patients with labral repair were restricted to 80° flexion for six weeks. The rehabilitation protocol after ACI consisted of continuous passive motion (CPM) therapy for four weeks (minimum three hours a day) and 20 kg partial weight bearing for six weeks postoperatively. Physiotherapy was allowed in pain-free range of motion. Low impact sport was administered at three months and high impact sport at six months postoperatively.

Clinical and outcome evaluation were performed at six weeks, three, six, 12 and 24 months after ACI. Additionally, data concerning pain medication, tolerability of the ACI and adverse events were collected.

### Statistical analysis

All statistical calculations have been done using SAS software, release 9.3 (SAS Institute Inc., Cary, NC, USA). For qualitative factors, absolute and relative frequencies are given. Quantitative variables are presented by mean ± standard deviation. In order to investigate changes over time, an analysis of variance (ANOVA) for repeated measurements has been performed for each quantitative outcome using the SAS procedure SAS MIXED (with patients’ ID as a random factor). Dunnett’s test has been applied in order to enable comparisons to baseline. Mean values between two groups (i.e., MACT (maximum achievable control technology) product) have been compared with two-sample *t*-tests. Furthermore, Pearson correlation coefficients have been assessed in order to quantify the strength of correlation between two quantitative parameters (i.e., between an outcome variable and cartilage defect size or size of labrum tear). A statistical test result has been considered as statistically significant for *p* < 0.05.

## Results

### Baseline characteristics

A total of 29 patients (27 men/two women) aged between 18 and 45 years (mean age 30.3 years) were included in this investigation. The average time of follow-up was 19 months (range 6–24 months). All patients were diagnosed with a full-thickness chondral defect of the acetabulum grade 3A–3D (nine 3A; eleven 3B; six 3C; three 3D) according to ICRS classification. Five patients showed radiographic osteoarthritis grade 2°, six grade 1° and 18 patients showed no signs of osteoarthritis.

All defects were located on the acetabulum between 12 and 5 o’clock position. The mean defect size was 2.21 cm^2^, no concomitant cartilage defect was seen. Twenty defects were caused by CAM-type FAI, six by combined FAI, one by Pincer-type FAI and two were of traumatic cause. No previous operation was performed on the same hip.

During index arthroscopy, 28 patients received additional treatment on the affected hip: offset reconstruction was performed on all 28 patients, of which 12 patients received additional acetabuloplasty.

Twenty-six patients had labral pathology of which 23 received labral repair with one to five anchors and three partial trimming of the labrum. The extension of the labral defects on a clock rating varied from 1 to 5 o’clock. The demographic data and baseline characteristics of the study population are illustrated in Table 1.

**Table 1.** Demographic data and baseline characteristics of the study population.

Demographics		(%)
Age in years	30.3 ± 6.9	(18–45) range
Gender (male/female)	27/2	(93/7)
Locality/grade of defect		
Acetabular	29	(100)
3A	9	(31)
3B	11	(38)
3C	6	(21)
3D	3	(10)
Grade of osteoarthritis		
0°	18	(62)
1°	6	(21)
2°	5	(17)
Type of FAI		
CAM	20	(69)
Pincer	1	(3)
Combined	6	(21)
Trauma	2	(7)
Total number of hips	29	(100)

### Functional outcome evaluation

The clinical efficacy of the MACI-System was evaluated with three validated outcome scales such as iHOT33, NAHS and Short-Form Health Survey. Each scale displayed a significant improvement (*p* < 0.05) at a mean of 16 months after ACI in comparison to baseline. The detailed illustration for each score is demonstrated in the following.

### International hip outcome tool (iHOT33)

All patients treated with ACI showed an overall improvement at 24 months according to the iHOT33 in comparison to baseline (*p* = 0.0002) (mean score at baseline: 48.9 ± 17.1%; 12 months after MACI: 70.1 ± 20.3% (19 patients); 24 months after MACT: 67.2 ± 23.2% (19 patients)).

At three months significant improvement was seen (*p* = 0.0007). There was further improvement at six months (*p* < 0.0001) and 12 months (*p* < 0.0001) after surgery.

### Non-arthritic-hip-score (NAHS)

Patients improved significantly in the Non-Arthritic-Hip-Score after the treatment with ACI compared to baseline at 24 months (*p* = 0.0009) (mean score at baseline: 66.6 ± 14.4%; 12 months after MACI: 79.4 ± 16.4% (19 patients); 24 months after MACT: 80.1 ± 11.6% (19 patients)). Significant improvement was monitored at six months (*p* = 0.0004) and 12 months (*p* = 0.0014) after surgery.

### Euro-Quol group score (EQ-5D)

Patients who underwent ACI of the hip showed an overall improvement according to EQ-5D when compared with baseline data (mean score at baseline: 58.7 ± 16.6; 12 months after MACT: 78.1 ± 16.8 (19 patients); 24 months after MACI: 73.1 ± 20.3). The improvements compared to baseline were significant (three months: *p* = 0.0003, six months: *p* = 0.0002; 12 months: *p* < 0.0001; 24 months: *p* = 0.0024).

All outcome evaluations are displayed in Figure 4.

### Subgroup analyses

**Figure 5.**
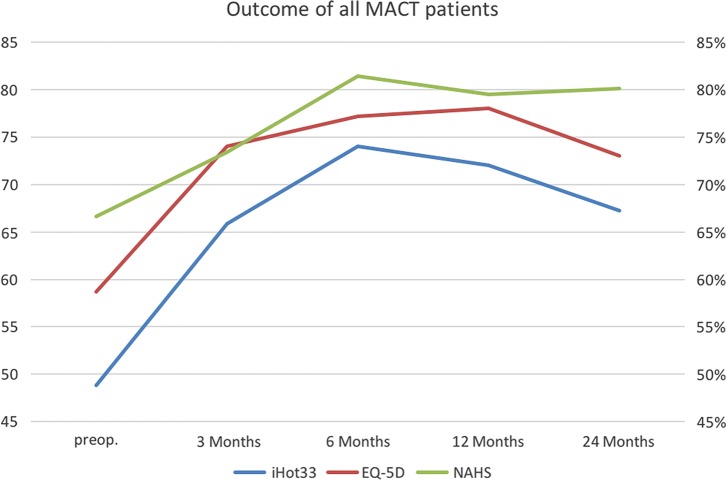
Outcome evaluation of all patients after MACI of the hip.

Three variables were categorized (cartilage defect size, labral defect size and MACI product) and analysed in regard to their potential influence on the outcome following arthroscopic MACI of the hip (Figure 5).

### Cartilage and labral defect size

Overall no relevant influence could be detected regarding the size of the cartilage defect and the size of the labral tear on the functional outcome in the iHOT33, EQ-5D and NAHS at 12 months and at 24 months (*p* > 0.05). All correlation coefficients have absolute values and are less than 0.3 due to the small number of patients. Therefore, no statistically relevant conclusions can be drawn.

### MACI product

The postoperative outcome results at 12 months (iHOT33 *p* = 0.4839; EQ-5D *p* = 0.6427; NAHS *p* = 0.2850) and 24 months (iHOT33 *p* = 0.4684; EQ-5D *p* = 0.4643; NAHS *p* = 0.4595) did not show a significant difference between the two MACI products using *t*-tests. Descriptive data of both products are illustrated in Table 2.

**Table 2.** Descriptive data on the two MACI products.

Product	Variable	*N*	*M*	*SD*	Min	Max	Variable	*N*	*M*	*SD*	Min	Max
Novocart	iHOT33 12 months	10	74.2	16.6	43.0	97.0	iHOT33 24 months	11	69.2	21.8	34.0	98.0
	EQ_5D 12 months	10	79.7	13.8	50.0	95.0	EQ_5D 24 months	11	76.1	15.4	40.0	95.0
	NAHS 12 months	10	84.0	10.8	62.5	100	NAHS 24 months	11	81.4	11.9	61.3	98.8
Codon	iHOT33 12 months	9	69.7	24.5	17.0	96.0	iHOT33 24 months	8	64.4	26.3	24.0	96.0
	EQ_5D 12 months	9	76.2	20.3	30.0	97.0	EQ_5D 24 months	8	68.9	26.3	35.0	97.0
	NAHS 12 months	9	74.4	20.6	23.7	88.8	NAHS 24 months	8	78.0	11.7	63.8	96.3

## Discussion

The aim of the present study was to report short-term results of 29 patients treated with ACI of the hip and a review of the current literature.

**Figure 6.**
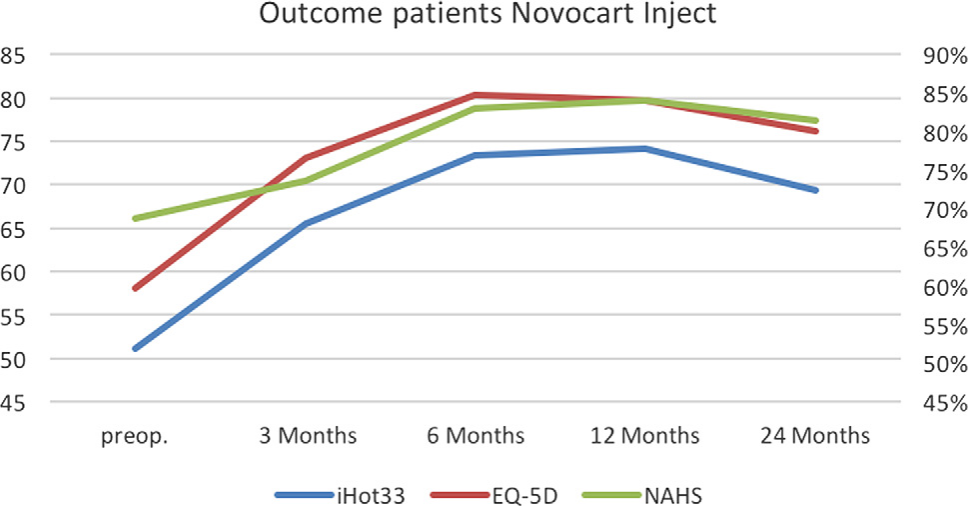
Outcome NOVOCART^®^ Inject.

**Figure 7.**
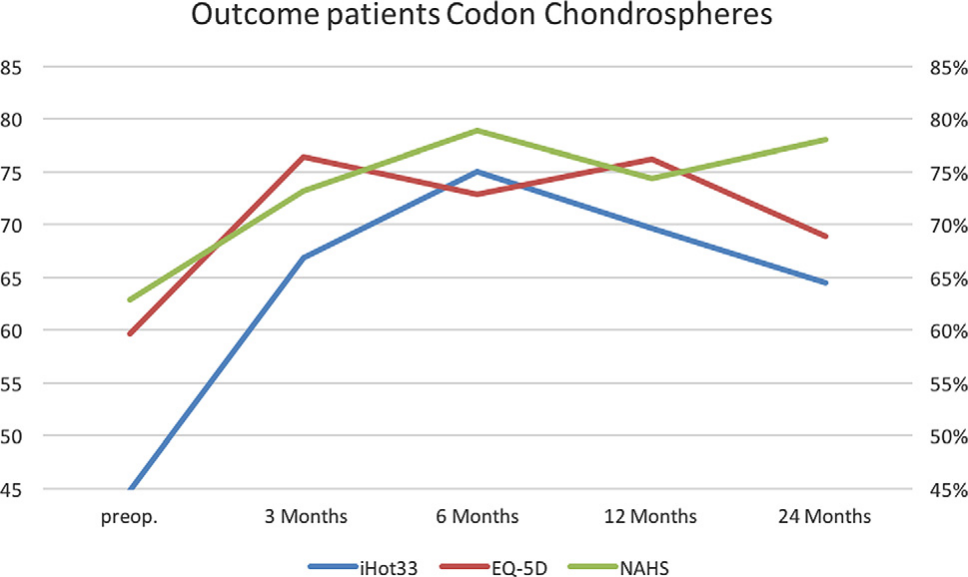
Outcome Chondrosphere^®^.

Two different MACI products were used, both can be transplanted arthroscopically (NOVOCART^®^ Inject/Chondrosphere^®^). The results were evaluated with respect to mental and physical health, pain and functionality in patients with isolated cartilage defects caused by FAI. In 19 cases, we were using NOVOCART^®^ Inject and Chondrosphere^®^ in 10 cases. Overall, the final scores after treatment with both products revealed statistically significant increased levels of activity and quality of life after an average follow-up of 16 months. However, we could not monitor significant differences between both products in the outcome scores. This indicates that in terms of pain relief and improvement of hip function the NOVOCART^®^ Inject (Figure 6) and Chondrosphere^®^(Figure 7) seem to be effective treatment methods for full-thickness cartilage defects of the hip.

The current literature indicates a correlation of cartilage defects and the degree of cartilage damage with inferior outcome results in FAI patients. In this context, Haviv et al. showed at a follow-up of 22 months that limited cartilage injury resulted in a better outcome in contrast to severe cartilage injury [3]. McCarthy et al. concluded that patients with the combination of severe cartilage injury (Outerbridge III° and IV°) and higher age have the highest risk for treatment failure “conversion to THA” [27]. Philippon et al. reported a correlation of the status of cartilage damage with the subjective outcome. At 2.3 years follow-up, cartilage status classified as mild, moderate and poor had modified Harris Hip Scores (mHHS) of 87, 79 and 62, respectively [4]. Several authors concluded that in cases of labral tears a coexisting cartilage defect seems to lead to significantly poorer outcome results [2, 5, 28]. These studies show that cartilage defects have the most direct impact on the postoperative outcome after hip arthroscopy. This indicates the importance of the appropriate treatment of cartilage defects of the hip.

In the hip, as a deep ball-and-socket joint is surrounded by a large number of muscles, access to the joint is difficult. The treatment of the central compartment is especially challenging, as joint distraction should be restricted to 10–15 mm and distraction times should be limited during arthroscopy to avoid complications [29]. The localization of cartilage defects of FAI patients, in the majority in the anterolateral position of the acetabulum, challenges cartilage repair systems to dispense with further fixation by suture or adhesives [15]. Accordingly, a self-adherent ACI-System whose applicator can easily be passed through an arthroscopic cannula and enables uniform distribution of chondrocytes in the defect area by a special vortexer seems to be the ideal solution for the hip joint [14].

To our knowledge, this is the first study reporting data about NOVOCART^®^ Inject for the treatment of cartilage defects in the hip. Preclinical research has proven positive biologic effects and biocompatibility of the components of NOVOCART^®^ Inject [18]. The transplant is characterized by stabilization of the chondrocyte phenotype [30] and the hydrogel has shown anti-angiogenic and anti-osteogenic effects that inhibit inflammation [19, 31].

The Chondrosphere^®^, an autologous MACI product, which can also be transplanted arthroscopically, has already been used in the hip joint [14]. This technique offers growth and phenotypic stability, while consisting of human autologous spheroids in 0.9% NaCl suspension. The spheroids derive from human autologous chondrocytes, which can produce cartilage-specific matrix and are able to build a three-dimensional structure under defined cell culture conditions [14, 26].

However, a possible advantage of NOVOCART® Inject may be the remarkable bonding capacity of the in situ polymerizable hydrogel. A comparison of both products is illustrated in Table 3.

**Table 3.** Comparison of the NOVOCART^®^ Inject and Chondrosphere.

NOVOCART^®^ Inject	Chondrosphere^®^
Cultivation within 3–4 weeks with pooled serum, BMP-2 and insulin	Cultivation within 6–7 weeks in 200 mL autologous serum
Injectable through a deformable needle	Injectable through a deformable applicator
Polymerized within 2 min after application through polyethylene glycol crosslinking	Bonding of chondrospheres after approximately 20 min
Characterized autologous chondrocytes and in situ polymerizing albumin-hyaluronic acid gel, phenotypic stability, positive biologic effects (Inhibition of alkaline phosphatase, vascularization, neurotrophic factors)	Complete autologous cell product, phenotypic stability, produces cartilage-specific matrix by induction of three-dimensional cell-cell contacts, biocompatibility
RCT at the knee running, studies with evidence level III at the knee	RCT at the knee running, multiple evidence level II and III studies at the hip, knee and ankle

Until now, three studies have displayed the feasibility and reported short-term data of Chondrosphere^®^ for use in the hip joint. Fickert et al. reported significant improvement in the outcome scores (mHHS and NAHS) in six patients with an average cartilage defect size of 3.5 cm^2^ at 11 months follow-up [14]. Körsmeier et al. included 16 patients with cartilage defects of 4.52 cm^2^ in their study. They monitored a significant improvement in the outcome scores at 16 months displayed in the Western Ontario and McMaster Universities Osteoarthritis Index (WOMAC) score and NAHS [16]. Schroeder et al. reported significant improvement in the iHOT33, mHHS and subjective hip value (SHV) scores in 20 patients with an average defect size of 5.05 cm^2^ at 12 months follow-up [17].

Fontana et al. in contrast used a polymer-based scaffold (BioSeed^®^-C, BioTissue Technologies GmbH) [15, 32], which was seeded with the expanded chondrocytes after cultivation. Thus it is not a classical MACI product, where scaffolds have to be seeded prior to cultivation. From the technical point of view, their rigid scaffold needed to be cut in approximate shape before introducing the folded scaffold through the cannula in the joint. Apart from the difficult handling, the scaffold is lacking in self-bonding capacity and as a result only acetabular defects could be treated arthroscopically without further fixation [15, 33].

However, Fontana et al. reported five-year follow-up results of 15 patients with chondral defects of the hip treated with ACI in comparison to a group with simple debridement in a retrospective study design. After 5 years, the ACI group showed a significant better outcome in the Harris Hip Score (HHS) compared to the debridement group [15].

In a second study, the same author compared 26 patients treated with ACI with 31 patients treated with an AMIC procedure (Chondro-Gide^®^, Geistlich Pharma AG, Switzerland). Both groups showed similar significant improvement in the mHHS at five-year follow-up in cartilage defects of 2.8 cm^2^ in the ACT-group and 2.9 cm^2^ in the AMIC-group [33]. However, both study results should be interpreted with care due to a possible selection bias as their control groups had been selected out of 144 patients.

Similar to this study, Fontana et al. excluded patients with osteoarthritis higher than grade 2° [15] since several authors reported that osteoarthritis may be a parameter to predict inferior results after hip arthroscopy in FAI patients [2, 34]. We excluded patients with corresponding cartilage defects “kissing lesions” as the results in the knee joint have been unsatisfying. This is in line with the finding of Fontana et al., who reported the worst results in patients with corresponding cartilage defects in the hip [15]. Schroeder et al. also excluded patients with radiographic signs of dysplasia. Patients with radiographic signs of osteoarthritis ≥ 1° according to Kellgren and Lawrence were also excluded [17]. Körsmeier et al. in comparison does not specify exclusion criteria [16]. An overview of the current literature is displayed in Table 4.

**Table 4.** Summary of the current literature after treatment of hip cartilage defects with ACI/MACI.

Author	Design	Procedure	Patients (male/female)	Mean age (range)	Follow-up (months)	Score improvement (pre-post)	Defect size (cm^2^)	Localization	Grade	Additional treatment
Fontana et al. [15]	Retrospective study	ACI, Polymer-based scaffold BioSeed-C (BioTissue Technologies GmbH)	15 (9/6)	40.7 (22–52)	73.8 (72–76)	HHS 39.4	2.6	A (*n* = 15) FH (*n* = 3)	Outerbridge 3–4	Not mentioned
Fickert et al. [14]	Case series	MACI, Chondrosphere (CO.DON^®^)	6 (5/1)	33 (25–45)	11.2	mHHS 23.5 NAHS 28.1	3.6	A (*n* = 5) FH (*n* = 1)	ICRS IIIA–IIB	LR 3 LPR 2
Körsmeier et al. [16]	Case series	MACI, Chondrosphere (CO.DON^®^)	16 (14/2)	31.8 (20–47)	16.1 (10–29)	NAHS 26 WOMAC 33	4.5	A (*n* = 16)	Outerbridge 3–4	LR 2 LPR 4
Schroeder et al. [17]	Case series	MACI, Chondrosphere (CO.DON^®^)	20 (16/4)	33 (22–49)	12.1 (6–24)	mHHS 30 iHOT33 35% SHV 22%	5.1	A (*n* = 20)	Full-thickness defects	LR 18
Fontana and de Girolamo [33]	Case series	ACI, Polymer-based scaffold BioSeed^®^-C (BioTissue Technologies GmbH) vs. AMIC Chondro-Gide^®^, (Geistlich Pharma AG, Switzerland)	ACT 26 (12/14) AMIC 31 (13/18)	ACT 36 ± 9.3 AMIC 36.4 ± 10.3	60	ACT mHHS 37.8 ± 5.9 AMIC mHHS 39.1 ± 5.9	ACT 2.8 ± 0.7 AMIC 2.9 ± 0.8	ACT A (*n* = 26) AMIC A (*n* = 31)	Outerbridge 3–4	Not mentioned

LR = Labrum Repair; LPR = Partial Resection Labrum; A = Acetabulum; FH = Femoral Head.

In the present study with an average defect size of 2.21 cm^2^, we could also show a significant overall improvement in all scores at 16 months follow-up. We could not see inferior outcomes in correlation to larger defect sizes at 12 and 24 months after treatment, but this has to be interpreted carefully because of the small size of the study group.

Unfortunately, we are not able to comment on the quality of repair tissue or the defect filling after MACI as magnetic resonance imaging (MRI) or second-look surgery with histological or histomorphological analysis was not performed in this study.

There are two studies that performed second-look surgery. Körsmeier et al. performed second-look arthroscopy in two patients due to unsatisfactory range of motion 5 and 8 months after chondrosphere^®^ implantation [16]. They describe a good ingrowth of the transplanted chondrocytes, but further histological examinations have not been performed. Fontana et al. performed one second-look arthroscopy in a patient who underwent AMIC 13 months previously. Here, a satisfactory tissue quality with the fibrocartilage-like aspect was observed. Unfortunately, no ACI patient had second-look arthroscopy [33]. However, in our opinion functional outcome is more important than imaging studies as a patient with satisfying outcome results but poor MRI scoring would not be advised to have revision surgery.

The results discussed above are not directly comparable, as different outcome scores have been used. Among others, Körsmeier et al. used the WOMAC score, which is only validated for patients with osteoarthritis [16]. Fontana et al. only used the mHHS/HHS, which is limited to pain, movement and daily activities [15, 33]. These conventional scores do not reflect the expectations and aims of the mainly young and active patients with FAI. This demonstrates the limitation of the mHHS, which has been published previously [35]. Similar to Schroeder et al. [17] the iHOT33 was used in our study, which has been developed and validated especially for FAI patients to evaluate patient’s symptoms, functional and sports limitation as well as social, emotional and occupational limitations [36]. Furthermore, we correlated the functional outcome results with the general health of the patient (EQ-5D) to evaluate possible influences on the results [25].

Several studies have reported on the importance of the integrity of the labral seal and superior results of labral repair in comparison to a partial excision [4, 37–39]. Therefore, it seems to be important to preserve the labrum to sustain its function as a seal and therefore its positive effects on the joint lubrication and protection of the cartilage surfaces [38]. Out of 29 patients 26 showed labral tears. In 23 of 26 (88%) patients, a labral repair was performed. In three patients (12%), the labral tear could not be repaired due to the severity of the tear. In the present study, no influence of the size of the labral tear could be seen in regard to the postoperative outcome.

### Complications

In this study as well as in the current literature severe adverse events were not observed. Most complications are due to nerve affections like neuropraxia of the nervus pudendus or hypoesthesia of the forefoot because of traction. Only Körsmeier described reductions of the range of motion in two cases, reductions of the range of motion, which could possibly be related to the implantation surgery. We did not have re-operations or conversions to THA within the 19-month follow-up.

### Limitations

There are several limitations. First of all, the present study only displays a small number of patients with a possible selection bias as mainly FAI patients were included. Unfortunately, there is no control group; hence, care should be taken when drawing conclusions. Only a short-term assessment of 19 months was done. We did not perform second-look arthroscopies or postoperative MRI scans in order to be able to comment on the defect filling. Additionally, no histomorphological analysis of cartilage biopsies was done. Therefore, we are not able to comment on the quality of the cartilage repair.

## Conclusion

In this study, we present short-term data of new arthroscopic injectable matrix-associated, autologous chondrocyte implants as a treatment option for full-thickness cartilage defects of the hip. All patient-administered assessment scores demonstrated an increase in activity level, quality of life and reduction of pain after a 19-month follow-up. Further randomized controlled trails (RCTs) with comparison of natural history, bone marrow stimulation techniques and MACI of the hip have to confirm the results in long-term follow-up.

## Conflict of interest

The authors declare no conflict of interest in relation with this paper.

## Funding

The authors received no financial support for the research and/or authorship of this article.
